# Structural and Morphological Evolution for Water-resistant Organic Thermoelectrics

**DOI:** 10.1038/s41598-017-13726-0

**Published:** 2017-10-16

**Authors:** Hyeon Jin Oh, Jae Gyu Jang, Jong-Gyu Kim, Jong-In Hong, Jaeyun Kim, Jeonghun Kwak, Sung Hyun Kim, Seunghan Shin

**Affiliations:** 1 Department of Chemistry, Dankook University, Cheonan, Chungnam 31116 Republic of Korea; 2 Green Materials and Process Group, Korea Institute of Industrial Technology, Cheonan, Chungnam 31056 Republic of Korea; 3 Department of Chemistry, Seoul National University, Seoul, 08826 Republic of Korea; 4 School of Electrical and Computer Engineering, The University of Seoul, Seoul, 02504 Republic of Korea; 5 Department of Carbon Fusion Engineering, Wonkwang Univeristy, Iksan, Jeonbuk 54538 Republic of Korea

## Abstract

We systematically investigated the effect of 2,5-bis(2-hydroxy-3-methacryloyloxypropoxy)-1,4:3,6-dianhydro-sorbitol (Iso-GMA) with different concentrations on the structural and morphological evolution of poly(3,4-ethylenedioxythiophene):poly(styrene sulfonate) (PEDOT:PSS) containing a fixed volume of dimethyl sulfoxide (DMSO) to realize water-resistant organic thermoelectric devices. As an additive, Iso-GMA is a hydrophilic and crosslinking agent that can interact with PEDOT and PSS chains by hydrogen bonding and/or dipole-dipole- or dipole-charge-interaction. The Seebeck coefficient and power factor in the film incorporating 3.0 vol% DMSO and 0.8 vol% Iso-GMA were respectively 1.82 × 10^2^ and 1.53 × 10^5^% higher than those of the pristine PEDOT:PSS film without additives (DMSO and Iso-GMA). These results can be attributed to the self-assembled and crosslinked fibril networks with optimized phase separation, where the film has densely-packed PEDOT and highly lamellar-stacked PSS. Also, the reduced charge carrier concentration from the structural characteristics originated in the higher thermoelectric properties. We introduced the schematic illustration to understand the chemical bonding among the components and the morphological evolution according to the Iso-GMA concentration. The increased mechanical strength by the interchain stacking degree of PEDOT and the crosslinking of Iso-GMA facilitate the film remained in a water bath for 0.5 h without physical degradation, and sustain the thermoelectric properties during 12 h in humid conditons.

## Introduction

Conventional conducting polymers have never been regarded to be as compatible as inorganic semiconductors for thermoelectric power generation owing to their low thermoelectric conversion efficiency caused by rather low electrical conductivity^1–3^. With advances made in enhancing their electrical conductivity, conducting polymers have attracted extensive attraction. In particular, poly(3,4-ethylenedioxythiophene):poly(styrene sulfonate) (PEDOT:PSS) is one of the most widely studied conducting polymers as a thermoelectric active material because its electrical conductivity can be increased to over 4000 S cm^−1^ by increasing the interconnected conductive pathways^4,5^. Even though PEDOT:PSS with high electrical conductivity is a good candidate as a thermoelectric active material, its hygroscopic property readily deforms its films under high humidity atmosphere^6–8^. Deformation of a PEDOT:PSS film may cause it to not only lose its intrinsic conductivity but also destroy its capability for thermoelectric power generation, which can lead to significant problems with regard to stability and durability^9,10^. For instance, organic solar cells with PEDOT:PSS have poor stability when exposed to moisture because of the damage in the active layer from water molecules^11,12^. Some attempts have been made the film to be water-resistant, in which the hydrophilic PSS moieties have been substituted with hydrophobic- or crosslinking additives^13–15^. However, these methods showed trade-off relationship between the water-resistance and its performance mainly due to the electrical insulating properties of the additives^16,17^. The water-resistance and humid-stability of PEDOT:PSS that retains the required electrical conductivity for organic thermoelectric devices have yet to be studied.

In this study, we investigated the structural and morphological evolution for water-resistant organic thermoelectric devices by the addition of 2,5-bis(2-hydroxy-3-methacryloyloxypropoxy)-1,4:3,6-dianhydro-sorbitol (Iso-GMA) with different concentrations at the fixed volume of dimethyl sulfoxide (DMSO). The PEDOT:PSS film with 3.0 vol% DMSO and 0.8 vol% Iso-GMA simultaneously showed a substantial increase in Seebeck coefficient and power factor. Furthermore, we found that adding Iso-GMA into PEDOT:PSS/DMSO produced distinct fibril networks. This morphology improves the water-resistance/humid-stability without physical degradation and reduction of the thermoelectric performance compared to the pristine PEDOT:PSS film without additives (DMSO and Iso-GMA).

## Material and Methods

### Chemical compounds

Isosorbide, Allyl bromide, Potassium hydroxide (KOH), Tetrabutylammonium bromide (TBAB), 3-chloroperbenzoic acid (m-CPBA), Dichloromethane, Methacrylic acid (MAA), Hydroquinone were purchased from Aldrich and Triphenyl phosphate was purchased from TCI. All solvents and reagents used in this study were commercially available and used without further purification. Thin layer chromatography was performed over Merck silica gel 60 F254 on aluminum foil. Merck silica gel 60 was used for stationary phase in chromatographic separation or silica pad filtration. ^1^H and ^13^C NMR spectra were obtained from Brucker DRX 300 NMR spectrometer. For NMR spectra analysis, samples were prepared by dissolving in CDCl_3_.

### Instruments and sample characterization

The sheet resistance (*R*_*S*_) was measured with a four-point probe system, which was used to calculate the electrical conductivity (*σ*) with the equation *σ* = 1/(*R*_*S*_ × *t*). The thickness (*t*) of samples was measured with an alpha-step profilometer (Veeco, Dektak Stylus Profilometer)^18,19^. The surface morphology of samples were acquired by atomic force microscopy (AFM, Veeco, NanoScope IV) with tapping mode at a scan rate of 1 Hz under ambient atmosphere. X-ray diffraction patterns were obtained by a Bruker D8 advance diffractometer, with a Cu K_α_ X-ray source (40 kV, 40 mA). Raman spectra were recorded with a Horiba Jobin-Yvon LabRam Aramis spectrometer with 632.8 nm line of a He-Ne laser. The X-ray photoelectron spectroscopy (XPS) spectra were recorded using SIGMA PROBE Model. The Hardness was obtained using AFM nanoindentation with spring constant of the cantilever 375 N m^−1^, 70 KHz. The average hardness was obtained by measuring 10 different locations on each film. The thermovoltage generated by temperature difference between hot and cold region in the film was measured with a digital multimeter (Agilent 3458 A) as the temperature difference was checked by two type-K thermocouples connected to each electrode^19^.

## Results and Discussion

Iso-GMA was synthesized by substituting diol groups in isosorbide by etherification and subsequent epoxidation for diallyl isosorbide, where two methacrylate groups were introduced for crosslinking reaction^20^. We expected that hydrophilicity and relatively high dipole moment (7.85 D) of Iso-GMA would allow it to readily interact with PEDOT and PSS via hydrogen bonding and/or dipole-dipole- or dipole-charge-interaction (Figs S1 and S2). Therefore, the addition of Iso-GMA into PEDOT:PSS/DMSO would produce a different phase separation and the corresponding electrical properties. In addition, crosslinking methacrylate functional groups, which are located on both ends of the molecule, would provide the film with water-resistance to improve the environmental reliability in a rainy outdoor environment.

**Figure 1:**
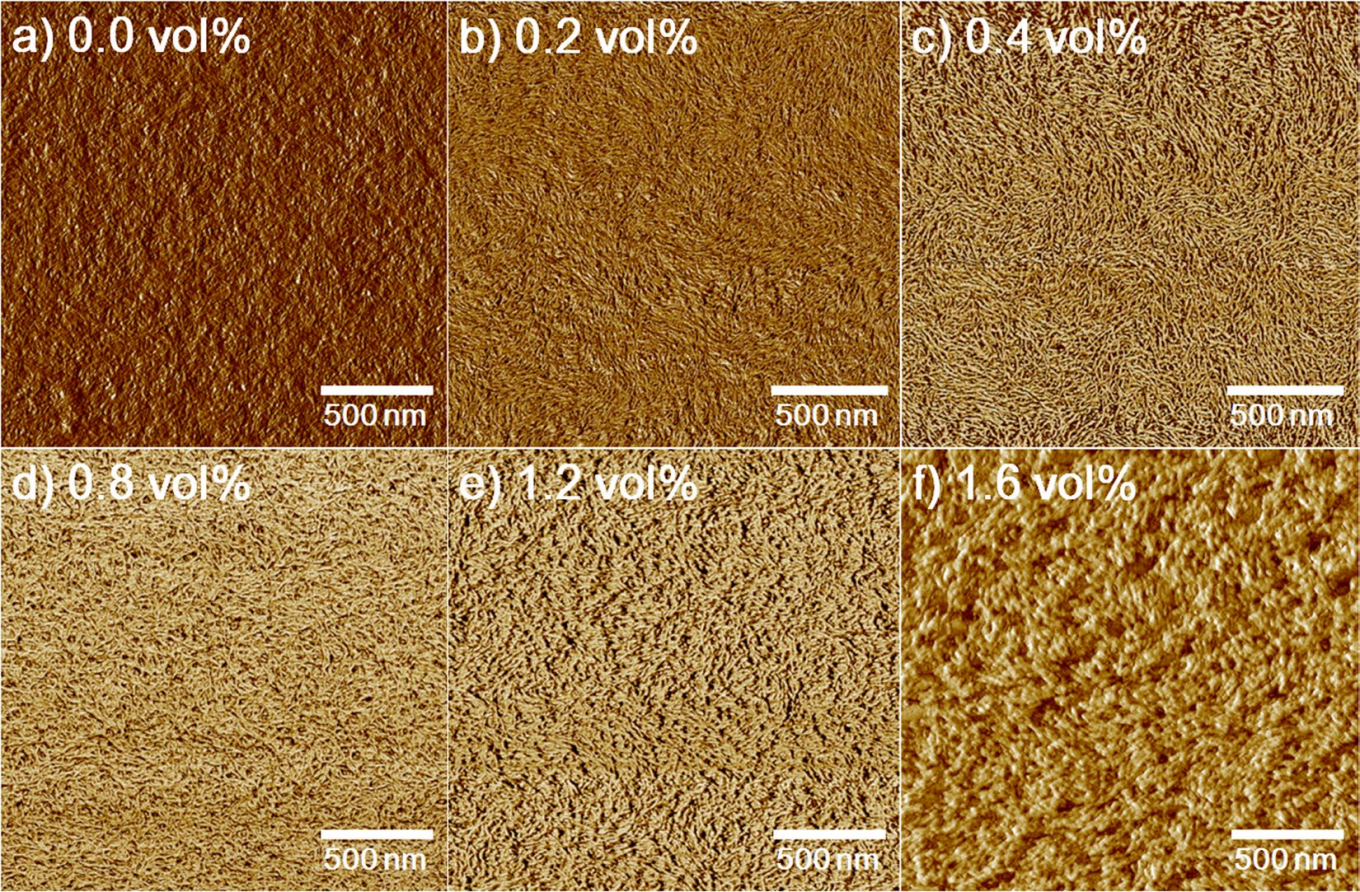
AFM phase images of the PEDOT:PSS/DMSO films with different Iso-GMA concentrations: (**a**) 0.0, (**b**) 0.2, (**c**) 0.4, (**d**) 0.8, (**e**) 1.2, and (**f**) 1.6 vol%.

In order to investigate the addition effect of Iso-GMA on the morphological evolution in PEDOT:PSS/DMSO, we used AFM to characterize the surface morphologies of the films, as shown in Figs 1 and S3. The volume of DMSO was kept constant at 3 vol% in all experiments. Because PSS chains typically consist of a few hundred monomer units, PEDOT and PSS grains were defined by PEDOT moieties surrounded by excess PSS molecules in the pristine PEDOT:PSS film without additives (DMSO and Iso-GMA) (Fig. S4)^21,22^. AFM images show rather disconnected bright and dark regions of PEDOT:PSS, which correspond to the PEDOT and PSS domains, respectively^23,24^. Compared to the pristine PEDOT:PSS film without additives (DMSO and Iso-GMA) in Fig. S4, Fig. S3a shows the increased grain size of the PEDOT when only DMSO was added. This variation was attributed to the change in the conformation due to the reduced coulombic interaction between PEDOT and PSS^25,26^. After Iso-GMA was added to PEDOT:PSS/DMSO, however, the surface morphologies of the films significantly changed. The size of the grains changed as the Iso-GMA concentration was increased (Fig. S3b–f). The average grain size of PEDOT increased up to 156.7 nm with 0.8 vol% Iso-GMA and then gradually decreased to 111.4 nm when more Iso-GMA was added (Fig. S3 and Fig. S5). In particular, fibril networks were clearly observed in the phase image when Iso-GMA was added (Fig. 1b–f). There is much more phase separation between PEDOT and PSS chains than that observed for the pristine PEDOT:PSS film without additives (DMSO and Iso-GMA). Thus, we deduced that the morphological evolution arises through significant phase separation of the PEDOT and PSS moieties induced by the interaction between Iso-GMA and PEDOT/PSS^27,28^. When Iso-GMA concentration was increased above 0.2 vol%, the thickness of the fibrils in the morphologies increased (Fig. 1). This may be attributed to the buckling between the fibrils from the increased crosslinking degree as Iso-GMA concentration was increased^29,30^. In addition, the interaction between Iso-GMA and PSS through hydrogen bonding may be another reason for the thickness change.

**Figure 2:**
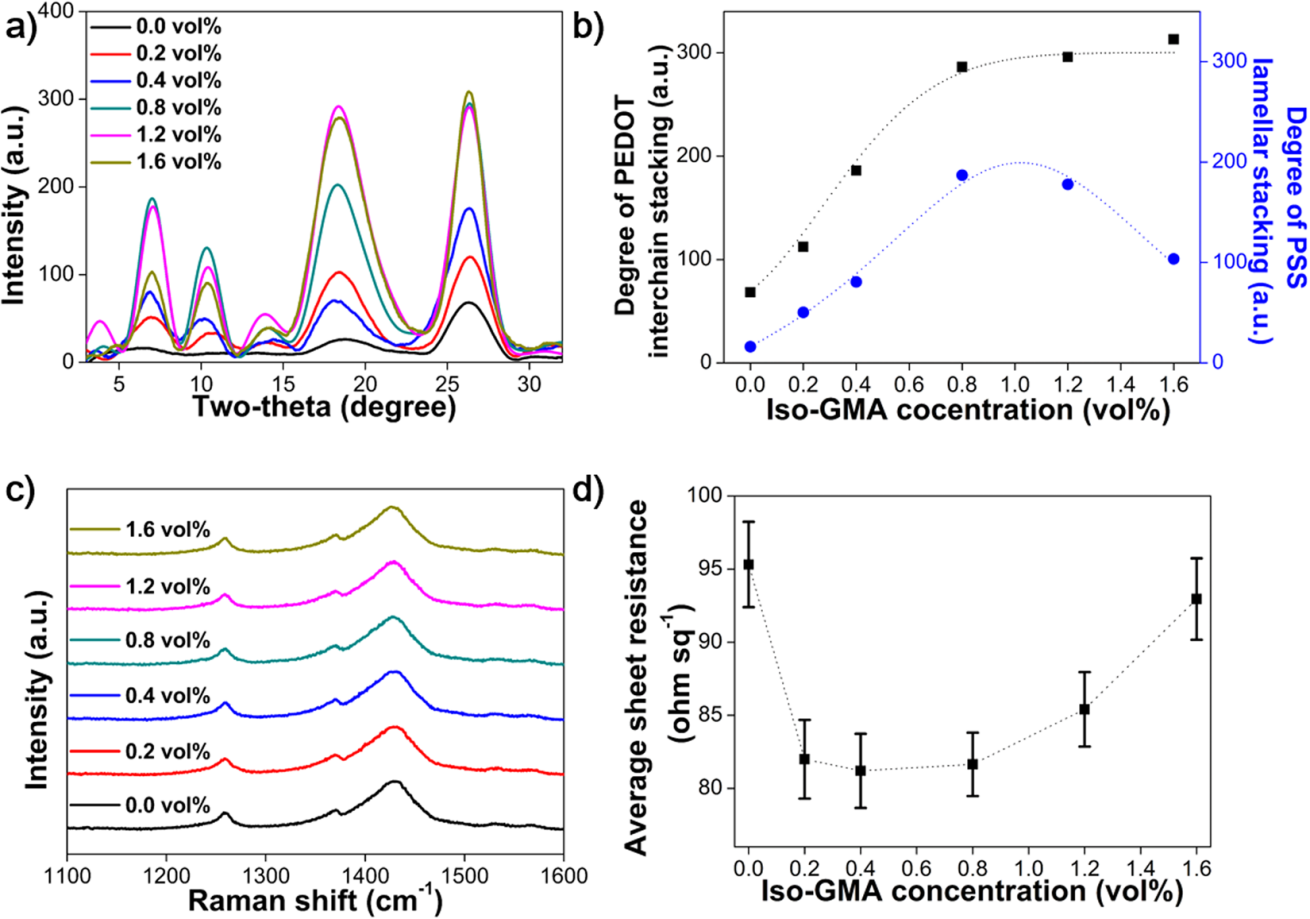
(**a**) XRD patterns, (**b**) interchain stacking degree of PEDOT and lamellar stacking of PSS, (**c**) Raman spectra, and (**d**) average sheet resistances of the PEDOT:PSS/DMSO films obtained with different Iso-GMA concentrations. The dotted line serves as a visual guide.

To achieve an in-depth insight into the structural characteristics of the self-assembled fibril morphology in the AFM images, we investigated XRD patterns of the PEDOT:PSS/DMSO films with different Iso-GMA concentrations, as shown in Fig. 2. All of the samples mainly showed two distinct peaks as the interchain stacking of PSS (2θ = 18.4°) and PEDOT (2θ = 26.1°)^31,32^. Fig. 2b shows the degree of the interchain stacking intensity for PEDOT (2θ = 26.1°) and the change of the lamellar stacking for PSS (2θ = 6.9°**)** with different Iso-GMA concentrations in PEDOT:PSS/DMSO^32^. Interestingly, the highest values were found when 0.8 vol% Iso-GMA was added. Above this Iso-GMA concentration, the variation for PEDOT changed little up to 1.6 vol% while the degree of PSS lamellar stacking gradually decreased. These results revealed that the 0.8 vol% Iso-GMA is the optimum concentration to induce both densely-packed PEDOT and lamellar-stacked PSS chains in the self-assembled fibril network.

We used Raman spectroscopy to investigate the resonant structural changes in PEDOT with different Iso-GMA concentrations in the PEDOT:PSS/DMSO films (Fig. 2c). The characteristic Raman peak for the PEDOT without Iso-GMA was located at 1430 cm^−1^. This was assigned to the *C*_*α*_ = *C*_*β*_ stretching vibrations of the five-member ring of PEDOT and shifted to 1426 cm^−1^ with 1.6 vol% Iso-GMA^33^. This shift represents that the structure of the resonant PEDOT changed from benzoid to quinoid^34^. Thus, this change in the PEDOT may improve the electrical properties corresponding to the resonant PEDOT structure^35,36^.

In order to determine how Iso-GMA affects the electrical properties of the PEDOT:PSS/DMSO films, we evaluated the sheet resistance for the films with different concentrations of Iso-GMA, as shown in Fig. 2d. The average sheet resistance was obtained with 4-point probe method by measuring 10 different locations on each film at room temperature. All the PEDOT:PSS/DMSO films maintained the average thickness of 110 nm except 170 nm of the 1.6 vol% Iso-GMA loaded film. The sheet resistance decreased from 95.23 to 81.60 ohm sq^−1^, when Iso-GMA concentration was increased from 0.0 to 0.8 vol%. This corresponded to the tendency for the interchain stacking degree of PEDOT in the XRD intensity (Fig. 2b,d). This result demonstrates that the morphological rearrangement of PEDOT and PSS by the addition of Iso-GMA facilitates the highly interconnected PEDOT chains inside the fibril network, providing better pathways for charge transport^5,37,38^. When Iso-GMA concentration was increased from 0.8 to 1.6 vol%, the sheet resistance increased from 81.60 to 93.00 ohm sq^−1^. In this region, the interchain stacking degree of PEDOT was almost constant, but the fibril thickness of PEDOT:PSS increased (Figs 1d–f and 2a, and S3d-f). Based on these results, we inferred that further phase separation of PEDOT:PSS does not appear, and the thicker fibrils due to Iso-GMA strongly suppress charge transport because it hinders the charge hopping between PEDOT chains^39^.

**Figure 3:**
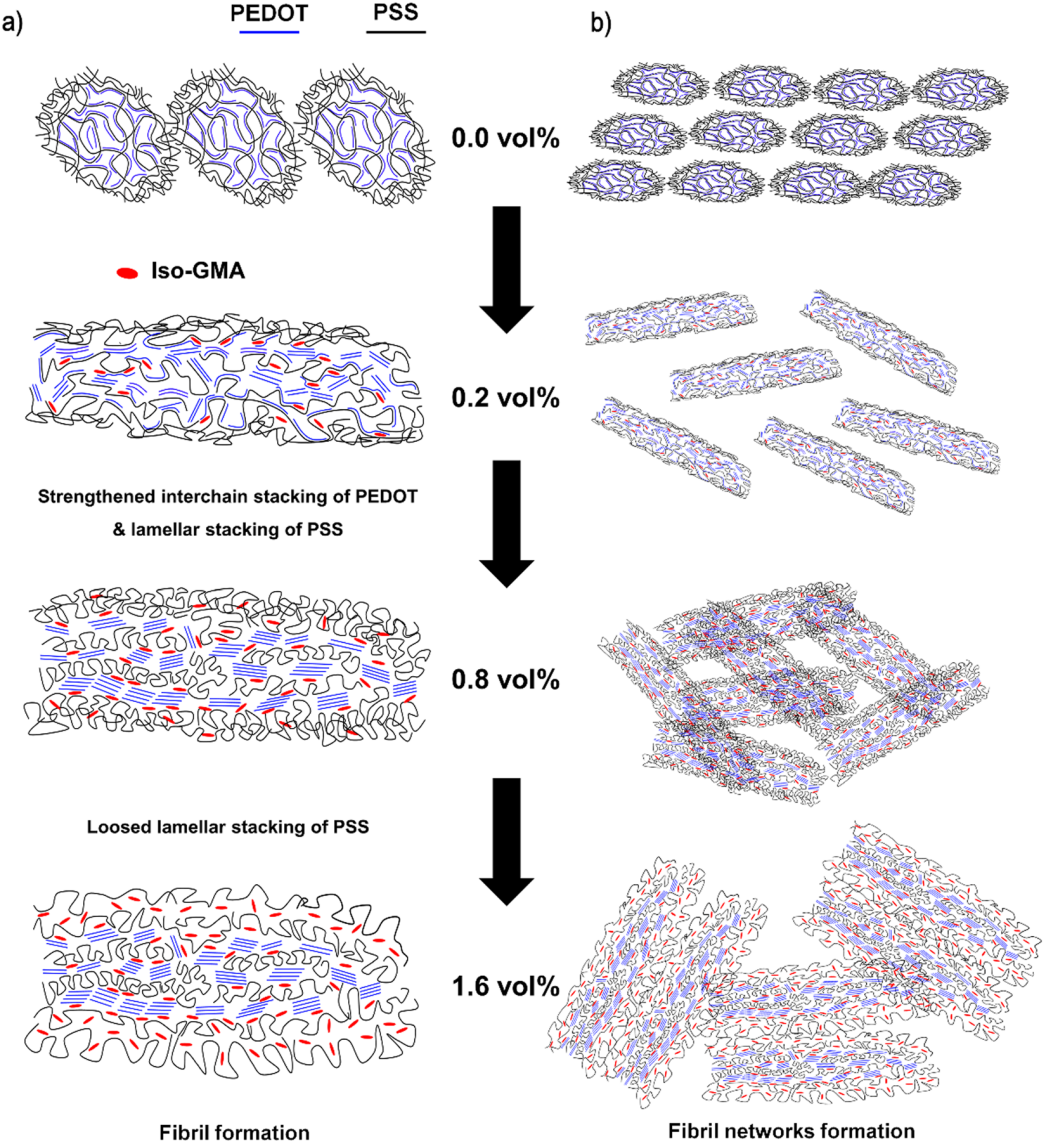
Schematic illustration of (**a**) the fibril and (**b**) fibril networks formation in the PEDOT:PSS/DMSO with different Iso-GMA concentrations.

Based on the above analysis of the AFM images, XRD patterns, and Raman spectra, we propose the mechanism for the self-assembled morphological evolution that forms fibril networks (Fig. 3). The Iso-GMA is a hydrophilic crosslinking agent which is composed of an isosorbide as a core moiety and two methacrylate groups attached at the terminal position. The addition of Iso-GMA to PEDOT:PSS/DMSO shows a highly crystalline fibril that consists of lamellar-stacked PSS and densely-packed PEDOT chains. An additive with a high dipole moment is known to easily reduced columbic interaction between PEDOT and PSS^35,36^. Because of the higher dipole moment of Iso-GMA than that of DMSO, adding it readily induces phase separation between PEDOT and PSS. The oxygen (–O–) and hydroxyl (–OH) groups of Iso-GMA can induce hydrogen bonding and/or dipole-dipole- or dipole-charge-interaction with PEDOT and PSS (e.g., OH···SO_3_^−^ (PSS^−^), OH···SO_3_H (PSSH), O···SO_3_H (PSSH), O···S^+^ (PEDOT) (Fig. S6)^28,35,36^. Compared with low boiling-point organic solvents, crosslinked Iso-GMA molecules reside in the resultant PEDOT:PSS/DMSO film even after the post-annealing process for film formation^28,40^. Thus, we infer that Iso-GMA with the interactions leads to anisotropic molecular rearrangement of PEDOT and PSS moieties, resulting in the fibril networks formation. The thickness of the fibrils in the film gradually increases with increasing Iso-GMA concentration. This may be because Iso-GMA readily interacts with PSS molecules rather than PEDOT moieties due to hydrogen bonding, which increases the thickness of the fibrils^41,42^. In addition, the buckling between the PEDOT:PSS fibrils may be another origin^29,30^. The relationship between the buckling and the phase separation can be understood by the intensity of the interchain stacking of PEDOT depending on the Iso-GMA concentrations in the XRD characteristic peaks. The value in the interchain stacking intensity for PEDOT increased up to 0.8 vol% Iso-GMA. Above 0.8 vol% Iso-GMA, the intensity was saturated, which means that phase separation between PEDOT and PSS was suppressed. Consequently, Iso-GMA predominantly participates in the phase separation where there is less than 0.8 vol% Iso-GMA. From this concentration of Iso-GMA, the buckling rather than the phase separation mainly occurs.

**Figure 4:**
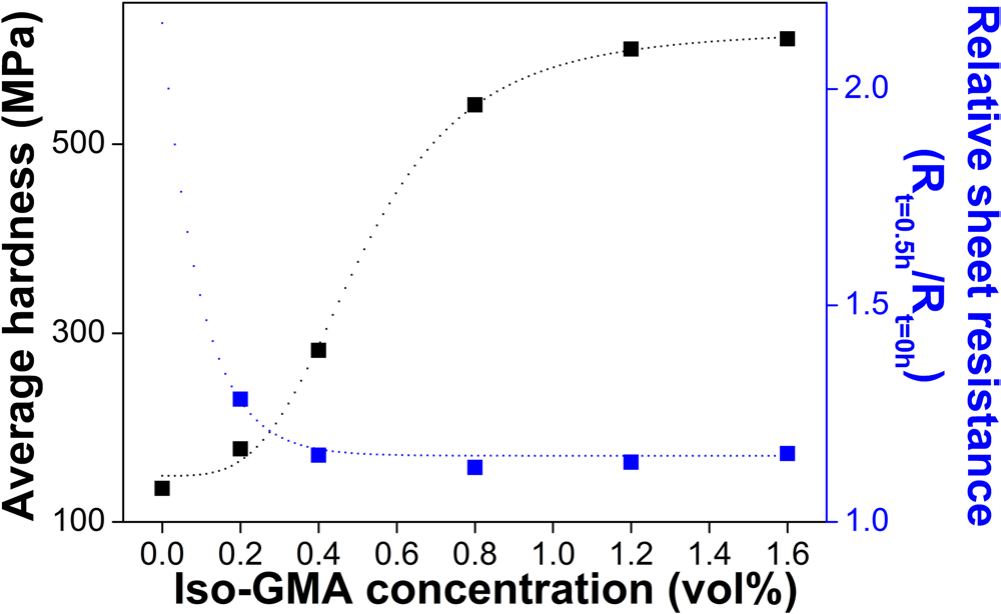
Hardness and relative sheet resistance of the PEDOT:PSS/DMSO films with different Iso-GMA concentrations. The t is sample storing time in humidity chamber. The dotted line serves as a visual guide.

In order to evaluate the mechanical durability of the PEDOT:PSS/DMSO films with different concentrations of Iso-GMA as a reflection of water-resistance, we performed nanoindentation experiments on the films. Figure 4 showed the mechanical hardness in the films with different Iso-GMA concentrations. The average hardness gradually increased with increasing Iso-GMA concentration. In particular, the film was about two times harder with 0.8 vol% Iso-GMA than with 0.4 vol% Iso-GMA. Above 0.8 vol% Iso-GMA, the variation in the mechanical properties were almost saturated. These distinct values were attributed to the formation of the crosslinked, self-assembled fibril networks, which corresponds to our hypothesis. When PEDOT:PSS/DMSO with Iso-GMA is annealed for thin film formation, buckling between the fibrils in the film can improve the mechanical properties^20^. The mechanical property is also related to the degree of the packing density in the self-assembled PEDOT and PSS^43,44^. Thus, the crosslinked PEDOT:PSS/DMSO with Iso-GMA maintained the tightly-bonded fibril networks, which hindered water uptake and swelling. The water-resistance of a film is a concept to evaluate the durability of polymer film after dipping in water^15^. To confirm the effects of Iso-GMA effects on water-resistance of PEDOT:PSS/DMSO films, we evaluated the relative sheet resistance according to Iso-GMA concentration. The sheet resistance of the films was measured before and after they were dipped in the water bath for 0.5 h (Fig. 4). The film with 0.0 vol% Iso-GMA was not estimated because it was out of the detection range in our measurement system. The variation (*R*_*t*=0.5h_/*R*_*t*=0_) of the relative sheet resistance showed a similar tendency to those of the hardness, which is inferred that the mechanical strength affected the water-resistance. Figure S8 showed the degree of film deformation with different Iso-GMA concentrations for 0.5 h. When the film was dipped in a water bath for 0.5 h, the water clearly dissolved the pristine PEDOT:PSS film without additives (DMSO and Iso-GMA). The other samples with 0.0, 0.2, and 0.4 vol% Iso-GMA were deformed and then degraded. However, the PEDOT:PSS/DMSO film with 0.8 vol% Iso-GMA retained the film formation. These results indicate that the mechanically strengthened PEDOT:PSS/DMSO film by the addition of Iso-GMA can provide humid-stability under high humid conditions.

**Figure 5:**
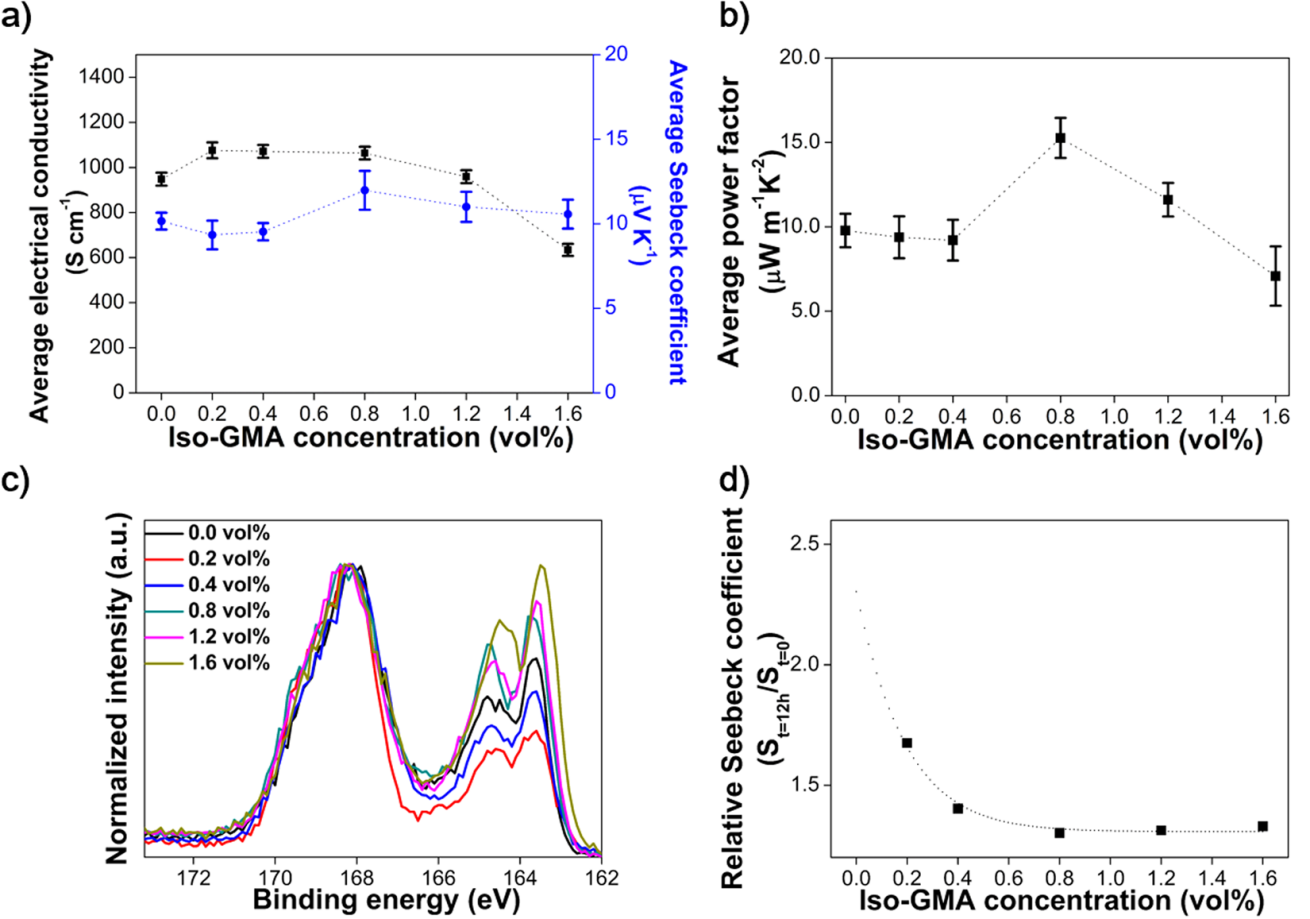
(**a**) Average electrical conductivities and Seebeck coefficients, (**b**) average power factors, (**c**) XPS spectra, and (**d**) relative Seebeck coefficient of the PEDOT:PSS/DMSO films with different Iso-GMA concentrations. The t is sample storing time in humidity chamber. All average values were obtained by 10 different locations and samples for conductivity and Seebeck coefficient/power factor, respectively.

The PEDOT:PSS/DMSO films with different Iso-GMA concentrations for organic thermoelectric devices were fabricated and measured their performances according to our previous method^19^. The thermoelectric properties consisted of Seebeck coefficient and power factor^45^. The power factor is given by *S* ^2^ *σ* where *S* and *σ* are the Seebeck coefficient and the electrical conductivity^45^. Figure 5a,b represent the average values of the conductivity and Seebeck coefficient, and power factor for the PEDOT:PSS/DMSO films with different Iso-GMA concentrations. The Seebeck coefficient of the film was highest at 0.8 vol% Iso-GMA and then decreased with more than 0.8 vol% Iso-GMA, which is known to be inversely proportional to the charge carrier concentration^7,18,46^. Figure 5c shows XPS data used to evaluate the charge carrier concentration in the PEDOT:PSS/DMSO films with different Iso-GMA concentrations^47–49^. PEDOT:PSS has two different sulfur atoms (S_2p_): one in the sulfonate group (166–170 eV) of PSS and the other in the thiophene group (162–166 eV) of PEDOT. The relative ratio of PEDOT to PSS at 0.4 vol% Iso-GMA decreased in comparison with that of the 0.0 vol% Iso-GMA sample. This implies that the charge carrier concentration increased, which indicates the reduced Seebeck coefficient and increased electrical conductivity. The 0.8 vol% Iso-GMA sample showed an increase in the Seebeck coefficient up to 11.98 μV K^−1^ with an electrical conductivity of 1063 S cm^−1^, which is consistent with the previous performance trend reported in the literature^50,51^. The increased Seebeck coefficient was ascribed to the dramatic decrease in the charge carrier concentration in comparison with that of the 0.4 vol% Iso-GMA. In detail, electrical conductivity is described as *σ = e·N·μ* where *e*, *N*, and *μ* are the electron charge, charge carrier concentration, and charge mobility, respectively^52^. Based on the above equation, the electrical conductivity is proportional to the charge carrier concentration. However, the 0.8 vol% Iso-GMA sample showed the opposite dependency. This unique enhancement of the electrical conductivity with 0.8 vol% Iso-GMA could be due to the densely packed PEDOT molecules with the interconnected fibril networks. This self-assembled, molecular rearrangement enhanced the charge mobility and the electrical conductivity^21^. For the PEDOT:PSS/DMSO film with more than 0.8 vol% Iso-GMA, the charge carrier concentration from the molecular ratio between PEDOT and PSS changed little but the Seebeck coefficient roughly decreased. This is because the increased thickness of the fibrils according to the crosslinking degree limited the charge transport, even though the electricmotive force is given by the temperature difference. As we described about the fibril formation, Iso-GMA may increase in the thickness of the insulating PSS moieties of the fibril by the buckling and/or hydrogen bonding. The power factor (15.26 μW m^−1^K^−2^) of the 0.8 vol% Iso-GMA sample provided an enhancement of 1.53 × 10^5^% compared to the value (0.01 μW m^−1^K^−2^) of the pristine PEDOT:PSS film without additives (DMSO and Iso-GMA) in Table S1.

To evaluate humid-stability of the PEDOT:PSS/DMSO thermoelectric devices with different Iso-GMA concentrations, the relative Seebeck coefficient (*S*_*t=12h*_/*S*_*t=0*_) was evaluated by comparison to the 0.0 vol% Iso-GMA sample (Fig. 5d). The humid-stability of device is a concept for device stability after exposure to humid conditions^53^. It is well known that Seebeck coefficient is increased when PEODT:PSS absorbs water from air^9,10^. The relative Seebeck coefficient in the sample with 0.0 vol% Iso-GMA was not obtained due to the detection limit in the sheet resistance. The value of *S*_*t=12h*_/*S*_*t=0*_ became stable more than 0.8 vol% Iso-GMA concentration, which is consistent with the tendency of *R*_*t=0.5h*_ */R*_*t=0*_ (Figs 4 and 5d). Consequently, the addition of Iso-GMA to PEDOT:PSS/DMSO significantly improves the water-resistance and humid-stability for outdoor applications in organic thermoelectric devices.

## Conclusions

The distinct morphologies in PEDOT:PSS were induced by different Iso-GMA concentrations with a fixed volume of DMSO (3.0 vol%), which could be attributed to the hydrogen bonding and/or dipole-dipole- or dipole-charge-interaction between Iso-GMA and PEDOT/PSS molecules. The addition of 0.8 vol% Iso-GMA to PEDOT:PSS/DMSO simultaneously enhanced the Seebeck coefficient of 1.82 × 10^2%^, the power factor of 1.53 × 10^5%^, and the electrical conductivity of 4.09 × 10^5%^ compared to the pristine PEDOT:PSS film without additives (DMSO and Iso-GMA). We believe that the enhanced thermoelectric properties are due to the structural and morphological evolution as the self-assembled and crosslinked fibril networks. In addition, the addition of Iso-GMA in PEDOT:PSS/DMSO films enhances the water-resistance and humid-stability.

## Electronic supplementary material

Supplementary Information
