# Supplementary material for: Structural and Morphological Evolution for Water-resistant Organic Thermoelectrics
Source: Sci Rep. 2017 Oct 16;7:13287. doi: 10.1038/s41598-017-13726-0 (PMC5643330; doi:10.1038/s41598-017-13726-0)
Supplement: Supplementary file 1 — Supplementary Information [file 41598_2017_13726_MOESM1_ESM.doc]

Supporting information

**Structural and Morphological Evolution for Water-resistant Organic Thermoelectrics**

Hyeon Jin Oh^a,b,‡^, Jae Gyu Jang^c,‡^, Jong-Gyu Kim^a^, Jong-In Hong^c^, Jaeyun Kim^d^, Jeonghun Kwak^d^, Sung Hyun Kim^*,e^, and Seunghan Shin^*,b^

a. Department of Chemistry, Dankook University, Cheonan, Chungnam 31116, Republic of Korea

b. Green Materials and Process Group, Korea Institute of Industrial Technology, Cheonan, Chungnam 31056, Republic of Korea.

c. Department of Chemistry, Seoul National University, Seoul 08826, Republic of Korea.

d. School of Electrical and Computer Engineering, The University of Seoul, Seoul 02504, Republic of Korea

e. Department of Carbon Fusion Engineering, Wonkwang Univeristy, Iksan, Jeonbuk 54538, Republic of Korea.

^*^Corresponding author (E-mails : shkim75@wku.ac.kr (S. H. Kim) and shshin@kitech.re.kr (S. Shin)), ^‡^ These authors contributed equally to this paper.

1. Synthesis of Iso-GMA

1.1 Isosorbide diallyl ether.

Isosorbide (3 g, 20.4 mmol), TBAB (300 mg, 0.94 mmol), and allyl bromide (15 ml, 173.55 mmol) was dissolved in a 50% aqueous NaOH (20 ml). The mixture was heated at reflux for 24 hours and then cool to room temperature. The resulting mixture was diluted with CH_2_Cl_2_. The organic phase was washed with water and dried over Na_2_SO_4_. Solvents were evaporated to afford a white liquid (3.5g, 76%). ^1^H NMR (300 MHz, CDCl_3_): δ 5.92-5.89 (m, 2H), 5.31 (m, 1H), 5.19-5.18 (m, 1H), 4.61 (m, 1H), 4.51 (m, 1H), 4.07 - 3.92 (m, 9H), 3.61-3.59 (m, 1H).

1.2 Isosorbide diglycidyl ether.

Isosorbide diallyl ether (3.5 g, 15.5 mmol) and m-CPBA (10.7g, 62 mmol) were dissolved in CH_2_Cl_2_. The reaction mixture stirred for 72 hours at room temperature. The organic solution was filtered to remove m-CPBA and washed with saturated sodium bicarbonate and distilled water several times to remove residue of m-CPBA. The organic phase dried over Na_2_SO_4_ and solvents were evaporated to afford a yellowish liquid (3.1 g, 77%). ^1^H NMR (300 MHz, CDCl_3_): δ 4.70-4.68 (m, 1H), 4.54-4.53 (m, 1H), 4.07-3.85 (m, 5H), 3.66-3.65 (m, 5H), 3.22-3.10 (m, 2H), 2.84-2.81 (m, 2H), 2.63 (m, 2H).

1.3 2,5-bis(2-hydroxy-3-methacryloyloxypropoxy)-1,4:3,6-dianhydro-sorbitol (Iso-GMA).

Isosorbide diglycidyl ether (3.10 g, 11.80 mmol), Hydroquinone (0.03 g, 0.28 mmol) and catalytic amount of triphenyl phosphate were stirred under N_2_. MMA (2.5g, 29.5 mmol) was added dropwise to the reaction mixture and heated at 100 ℃ for 5 hours. The reaction mixture extracted with CH_2_Cl_2_. The organic phase was washed with water and dried over Na_2_SO_4_. Solvents were evaporated to afford a crude liquid, which was purified by silica gel column chromatography with gradient elution from hexane to 80% ethyl acetate in hexane to give a yellowish liquid. (2.3 g, yield: 45 %). ^1^H NMR (300 MHz, CDCl_3_): δ 6.15 (s, 2H), 5.62 (s, 2H), 4.69 (s, 1H), 4.54 (s, 1H), 4.25 (m, 4H), 4.11 - 3.93 (m, 5H), 3.74 - 3.38 (m, 5H), 1.97 (s, 6H); ^13^C NMR (75.47 MHz, CDCl_3_): δ 171.0, 167.1, 135.8, 125.8, 85.8, 79.7, 75.2, 72.7, 70.0, 18.1.

**2. Sample preparation and device fabrication**

The commercial PEDOT:PSS (Clevios PH 1000 from H.C. Stark) was filtered through a 0.45 μm pore size hydrophilic filter. The glass substrates (2.5 cm × 2.5 cm) were cleaned with ultrasonic treatment in both distilled water and isopropyl alcohol, followed by drying in a vacuum oven for 12 h. The filtered PEDOT:PSS solution were mixed with DMSO or Iso-GMA/DMSO at various concentration and stirred by planetary mixture for 10 min. The cleaned substrates were treated with UV/ozone for 15 min and the formulated solutions were spin-coated at 1000 rpm for 60 s. Then, they were pre-annealed at 120 °C for 5 min and sequent post-annealed at 150 °C for 30 min to form crosslinkers.^1^ To fabricate the thermoelectric devices, Au electrodes of 100 nm were thermally evaporated on the PEDOT:PSS/Iso-GMA film through a shadow mask under a base pressure of < 10^-4^ Pa.^2^ Two Au electrodes with a length and width of 17 and 8 mm, respectively, were separated by a distance of 2 mm, as shown in Fig. S7. The two type-K thermocouples and current-voltage (I-V) probes were affixed to the Au electrode, 10 mm apart.

3. Humidity test

The homemade humidity chamber (30 cm x 30 cm x 30 cm) consisted of acryl-plates and high humid atmosphere controlled by a humidifier. We measured relative humidity by hygrometer, showing relative humidity 95 %. After storage in the humidity chamber, we measured the Seebeck coefficient and sheet resistance of PEDOT:PSS/Iso-GMA films.^3,4^

**4. Supporting figures**


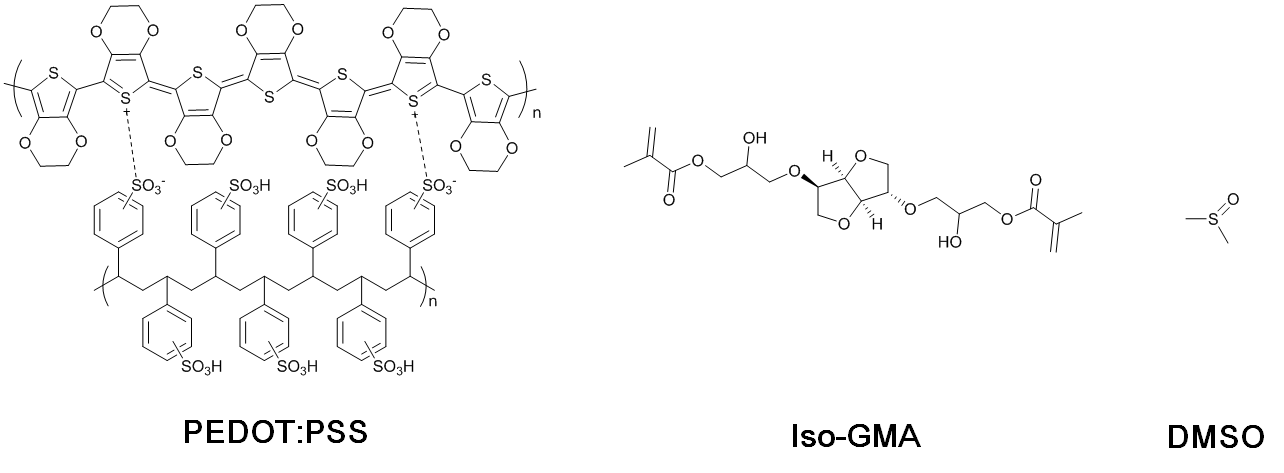


Fig. S1. Molecular Structures of poly(3,4 Ethylenedioxythiophene):poly(styrenesulfonate)(PEDOT:PSS), 2,5-bis(2-hydroxy-3-methacryloyloxypropoxy)-1,4:3,6-dianhydro-sorbitol (Iso-GMA), and Dimethyl sulfoxide (DMSO).


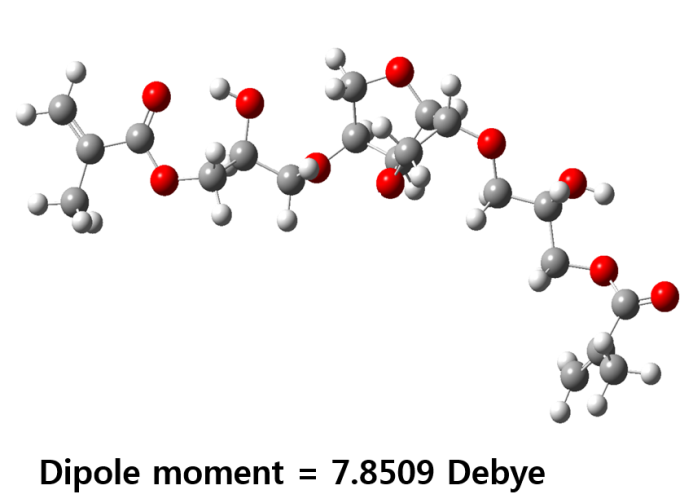


Fig. S2. Dipole moment of Iso-GMA.


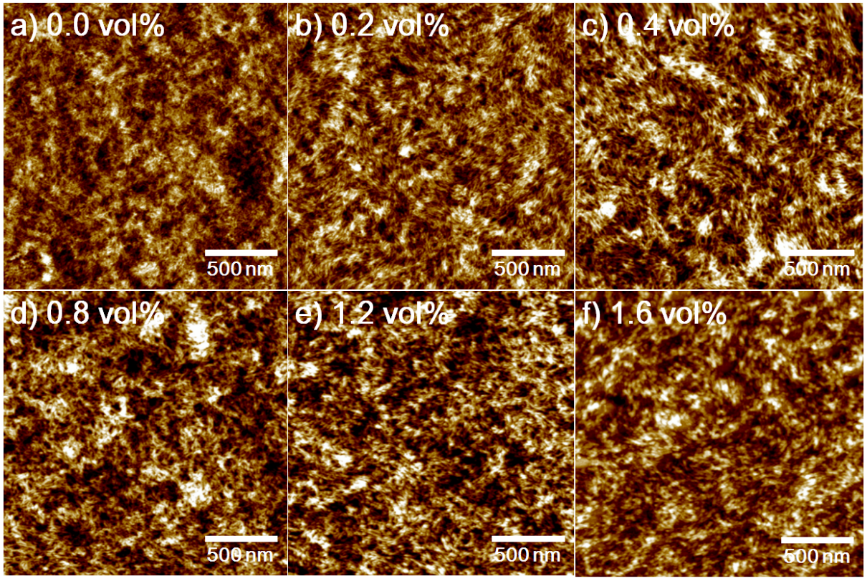


Fig. S3. AFM topographic images of the PEDOT:PSS/DMSO films with different Iso-GMA concentrations: a) 0.0, b) 0.2, c) 0.4, d) 0.8, e) 1.2, and f) 1.6 vol%.

**Fig. S4.** AFM images of topographic (left) and phase (right) images of the pristine PEDOT:PSS film without additives (DMSO and Iso-GMA).


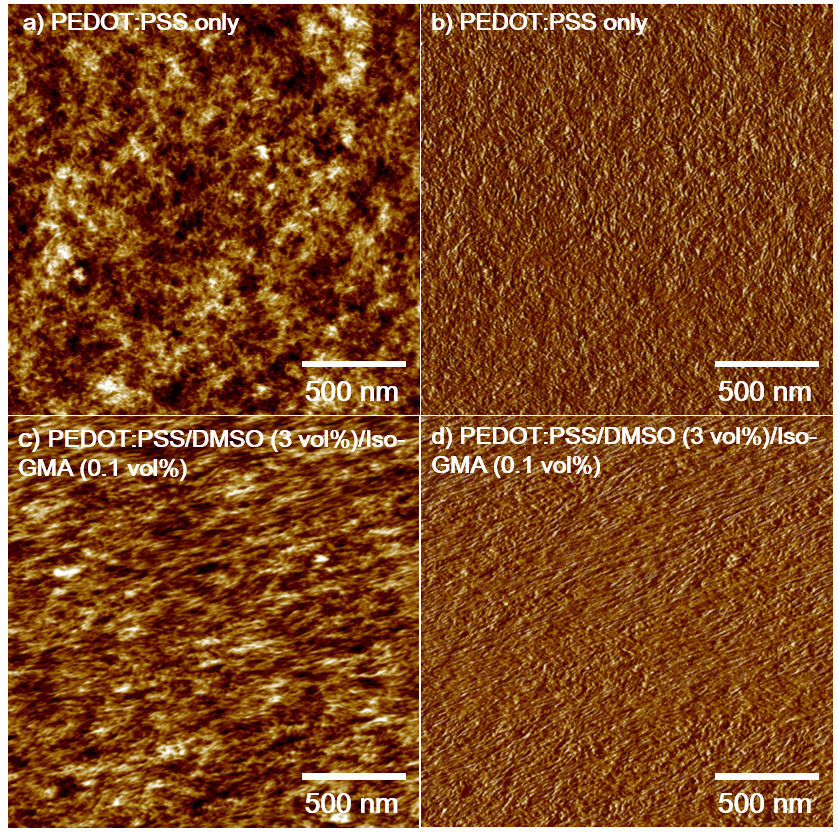


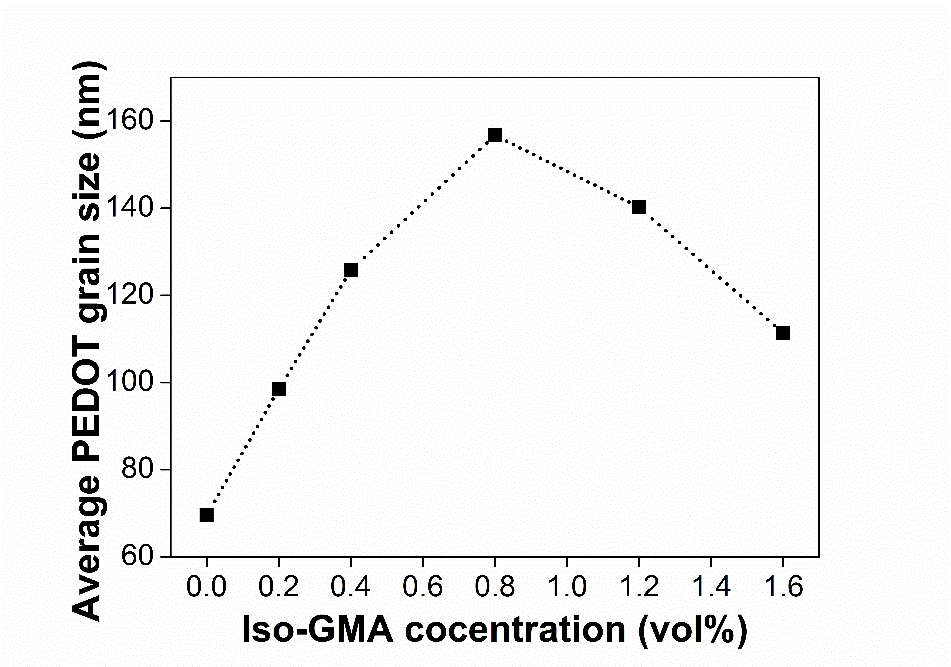


Fig. S5. Average grain size of PEDOT in the PEDOT:PSS/DMSO films with different Iso-GMA concentrations. The grain size was calculated by using particle analysis in the NanoScope Analysis software v. 1.40 from Bruker. The dotted line serves as a visual guide.


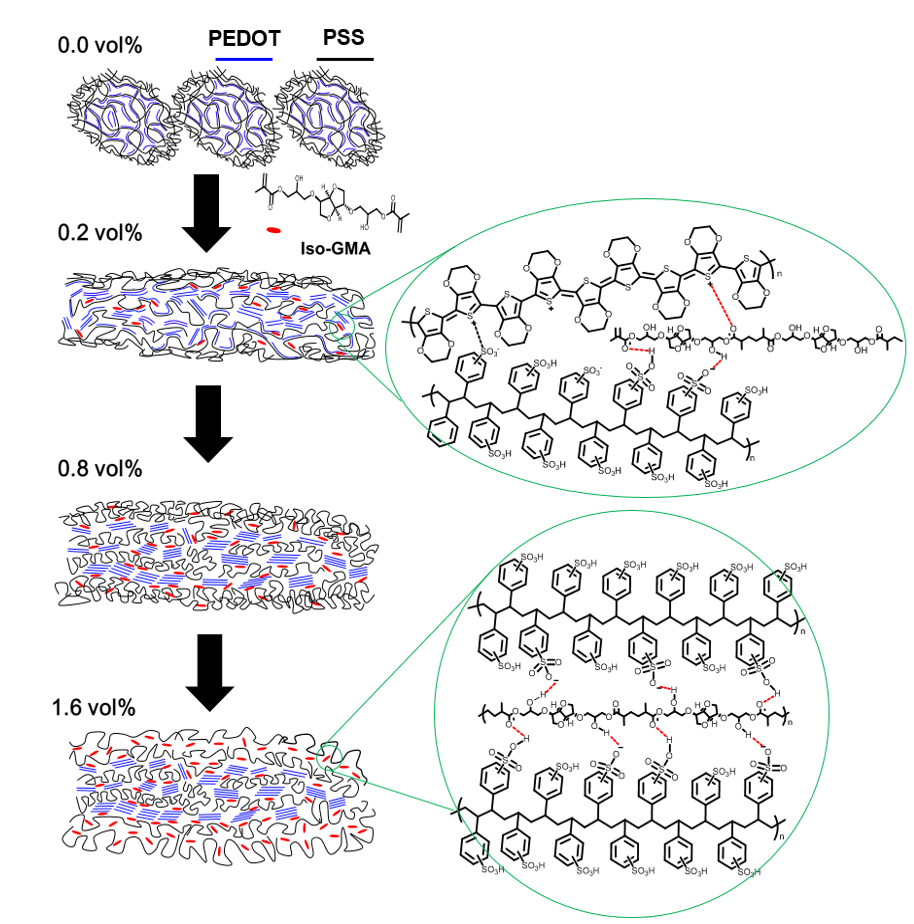


**Fig. S6.** Schematic illustration of hydrogen bonding and/or dipole-dipole- or dipole-charge-interaction with PEDOT and PSS in PEDOT:PSS/DMSO with different Iso-GMA concentrations.


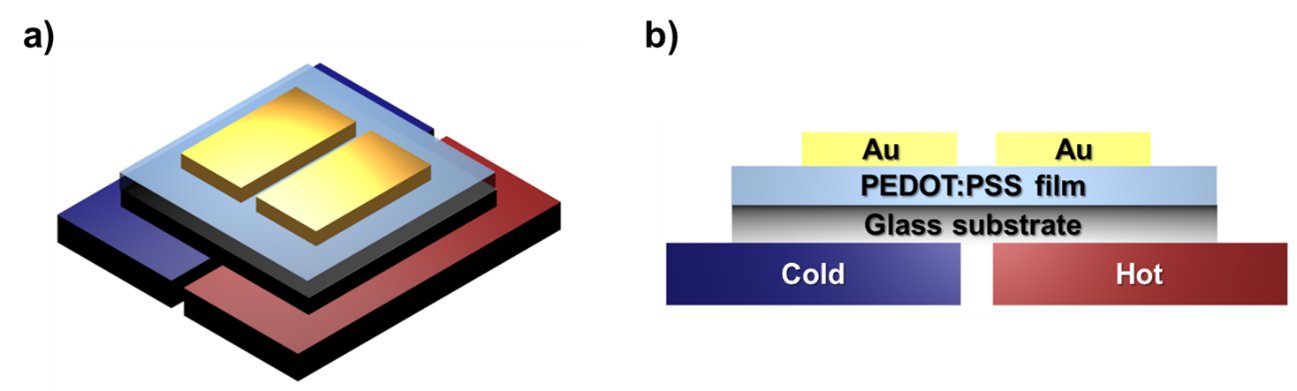


**Fig. S8.** Water-resistance tests of a) the pristine PEDOT:PSS film without additives (DMSO and Iso-GMA) and the PEDOT:PSS/DMSO films with the addition of b) 0.0, c) 0.2, d) 0.4, and e) 0.8 vol% Iso-GMA. The dotted red circles serve as a visual guide.


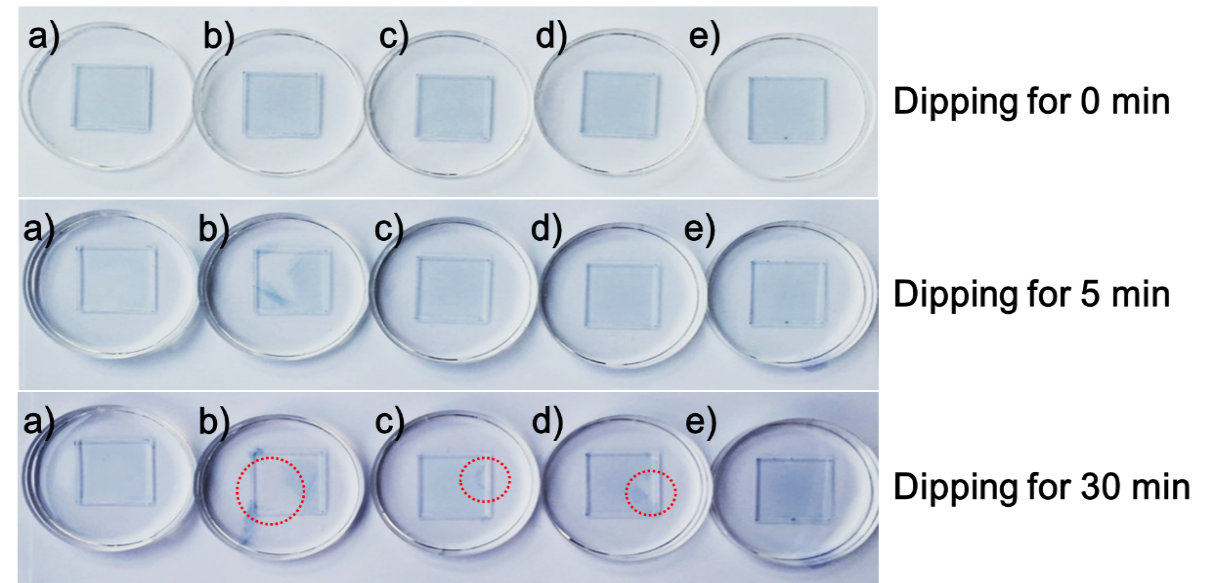


**Fig. S7**. Schematic illustration of thermoelectric device structure: a) top view and b) side view.

**Table S1.** The thermoelectric properties of the pristine PEDOT:PSS film without additives (DMSO and Iso-GMA) and the PEDOT:PSS/DMSO^a^ film with 0.8 vol% Iso-GMA.

| Sample | Thermoelectric properties | | | | | |
| --- | --- | --- | --- | --- | --- | --- |
|  | Electrical conductivity  (Scm^-1^) | | Seebeck coefficient  (μVK^-1^) | | Power factor  (μWm^-1^K^-2^) | |
|  | Value | Percent-age increase (%) | Value | Percent-age increase (%) | Value | Percent-age increase (%) |
| Pristine PEDOT:PSS film without additives (DMSO and Iso-GMA) | 0.26 | - | 6.58 | - | 0.01 | - |
| PEDOT:PSS/DMSO^a^  film with 0.8 vol% Iso-GMA | 1063.52 | 4.09 × 10^5^ | 11.98 | 1.82 × 10^2^ | 15.26 | 1.53 × 10^5^ |

^a^ The volume of DMSO was constant at 3.0 %.

**5. Reference**

1. Lukaszczyk, J., Janicki, B., Frick, A., Investigation on synthesis and properties of isosorbide based bis-GMA analogue, *J. Mater. Sci. Mater. Med.* **23**, 1149-1155 (2012).

2. Kim, J., Jang, J.G., Hong, J.I., Kim, S.H., Kwak, J., Sulfuric acid vapor treatment for enhancing the thermoelectric properties of PEDOT:PSS thin-films, *J. Mater. Sci. Mater. Electron.* **27**, 6122-6127 (2016).

3. Kim, G.-H., Kim, J., Pipe, K.P., Humidity-dependent thermoelectric properties of poly(3,4-ethylenedioxythiophene):poly(styrene sulfonate), *Appl. Phys. Lett.* **108**, 093301-093305 (2016).

4. Cho, W., Hong, J.K., Lee, J.J., Kim, S., Kim, S., Im, S., Yoo, D., Kim, J.H., Synthesis of conductive and transparent PEDOT:P(SS-co-PEGMA) with excellent water-, weather-, and chemical- stabilities for organic solar cells, *RSC Adv.* **6**, 63296-6330 (2016).
